# Hunting territories and land use overlap in sedentarised Baka Pygmy communities in southeastern Cameroon

**DOI:** 10.1038/s41598-021-83223-y

**Published:** 2021-02-10

**Authors:** Julia E. Fa, Guillermo Ros Brull, Eva Ávila Martin, Robert Okale, François Fouda, Miguel Ángel Fárfan, Bradley Cain, Rohan Fisher, Lauren Coad, Stephan M. Funk

**Affiliations:** 1 Department of Natural Sciences, School of Science and the Environment, Manchester Metropolitan University, Manchester, M1 5GD UK; 2 Center for International Forestry Research (CIFOR), CIFOR Headquarters, Bogor, 16115 Indonesia; 3 Zerca Y Lejos ONGD, c/Sambara 128, 28027 Madrid, Spain; 4 Departamento de Biología Animal, Facultad de Ciencias, Universidad de Málaga, Campus de Teatinos, 29071 Málaga, Spain; 5 Instituto IBYDA, Centro de Experimentación Grice-Hutchinson, Loma de San Julián 2, Barriada de San Julián, 29004 Málaga, Spain; 6 Research Institute for the Environment and Livelihoods, Charles Darwin University, Ellengowan Dr, Casuarina, NT 0810 Australia; 7 Department of Zoology, Interdisciplinary Centre for Conservation Science, University of Oxford Zoology Research and Administration Building, 11a Mansfield Road, Oxford, OX1 3SZ UK; 8 Nature Heritage, Channel Islands, Jersey

**Keywords:** Biodiversity, Conservation biology, Tropical ecology

## Abstract

A significant number of Baka Pygmies in Cameroon have been sedentarised in roadside villages, in contrast to their nomadic hunter-gatherer existence of the past. Although this change in lifestyle has had important consequences on health, most Baka villages still supplement their diets from forest products, especially wild meat. We used a combination of participatory methods and monitoring of individual hunters to map hunting territories in 10 Baka villages in southeastern Cameroon. From these, we determined whether wild meat extraction levels per village were related to the size of hunting territories, measured habitat use by hunters and finally defined the overlap between hunting territories and extractive industries in the region. Mapped village hunting areas averaged 205.2 ± 108.7 km^2^ (range 76.8–352.0 km^2^); all villages used a total of 2052 km^2^. From 295 tracks of 51 hunters, we showed that hunters travelled an average of 16.5 ± 13.5 km (range 0.9–89.8 km) from each village. Home ranges, derived from kernel utilization distributions, were correlated with village offtake levels, but hunter offtake and distance travelled were not significantly related, suggesting that enough prey was available even close to the villages. Hunters in all village areas exhibited a clear bias towards certain habitats, as indicated by positive Ivlev’s index of selectivity values. We also showed that all village hunting territories and hunter home ranges fall within mining and logging concessions. Our results are important for local understanding of forest land uses and to reconcile these with the other land uses in the region to better inform decisions concerning land use policy and planning.

## Introduction

Subsistence economies of indigenous peoples are based on the use of and access to natural resources. The protection of these resources and the traditional practices of resource utilization are essential to ensure the survival of these peoples. Convention 169 of the International Labour Office specifies that indigenous and tribal peoples have the right to participate in the use, management, protection, and conservation of natural resources, as well as the right to be consulted before natural resources on their territories are explored or exploited^1^. As MacKay^2^ points out, these rights pre-date the existence of any concessions the state may have granted indigenous peoples because they are derived from the ancestral occupation and traditional use of lands and resources by these peoples, as well as from their laws and traditional customs related to property and resource use.

The humid forests in the southern part of Cameroon are occupied by indigenous Baka Pygmy groups and Bantu-speaking swidden agriculturalists. The term Pygmy, although sometimes considered pejorative, is still widely employed in the literature, see^3^ to capture the diversity of societies that make up the rainforest hunter-gatherers of the Congo Basin^4^. Formerly strict hunters-gatherers, the Baka differ from the more sedentary neighbouring Bantu-speaking people with whom they maintain complex social, economic, and symbolic relations^5,6^. Pygmies primarily rely on a subsistence economy based on the exploitation of wild products, hunting of wildlife and gathering of plant resources^7–9^. Diverse hunting techniques are employed, and these are bound to their social structure and rituals^5,8,10–12^.

The Baka have witnessed the gradual reduction of access to forest resources due to competition from extractive industries and conservation initiatives^13^, and a shift from a nomadic to a predominantly sedentary existence. Until the mid-twentieth century, the Baka lived in small itinerant groups of 30–40 individuals in the forest^7^. However, during the twentieth century the adoption of agriculture and settlement along roads have modified their way of life, although they still exhibit high seasonal mobility associated to a specific forest habitat type; movements now maintained by shifting between settlement and forest camp-life^14–16^. Fixed settlement along roads was the result of the implementation of “development assistance” programs by the State after independence, as early as 1910 and then continuing slightly after 1960^15,17–21^; the reason given being that the settling down of nomadic peoples was a pathway to increased access to services^22^. As a result of resettlement programmes, numerous Baka groups in southern Cameroon now live in villages on the same road as Bantu‑speaking villagers/farmers as neighbours. Since at least the 1950s^23^ resettled Baka in our study area have engaged in subsistence agriculture by opening their own plots^15,24–26^. Sedentarised Baka groups, however, still depend on wild meat and other forest products for both their diet and income, often to different degrees^10,11,27–29^. Hunting studies of forest-living Pygmies throughout the Congo Basin show that mammals, especially ungulates and within this group duikers, are the main prey, and compared to non-Pygmy hunters they sell a very small proportion of animals hunted^30^.

As a result of sedentarisation many Baka have changed their mobility patterns^15,20,21,23^. Traditionally, Baka spatial organization would revolve around the various forest camps, which may change between seasons^21^. During the last decades, this organization has reoriented away from truly nomadic living to one of life between village settlement and forest camps^27^. This change in lifestyle has been associated with a marked decline in physical and mental health^31^. It has also had clear consequences on diets and food security, as shown by the healthier diets of Baka Pygmies living in isolated villages compared to those in villages closer to markets^28^. Research on some Baka groups in southeastern Cameroon indicate that although their food consumption patterns had changed after social transition, supplementing their life in the village with time in forest camps reduced stress and helped them to maintain a better nutritional status^32^.

Much of the Cameroonian humid forests are under increasing pressure from logging, mining and small-scale farming. Since the establishment of permanent villages along roads, agricultural fronts have expanded into the forest^33–36^. This has precipitated two key forms of conflict: competition for land between forest and agricultural use at the local scale, and between forest conservation and economic development at the landscape scale^37,38^. Although there have been studies on hunting and the use of wildlife for food in the region^8,10,12,39^, landscape-level analyses of natural resource use are noticeably absent for both plants and animals. Recent studies have described the spatial distribution and dynamic of hunters, hunts, prey, and hunting territories in the Amazon region, see^40,41^, but few have been undertaken in Africa (but see Kümpel et al.^42–44^). Understanding the spatial extent of hunting practices, the overlap of tenure systems and the scales at which land management operates is fundamental to developing and assessing the sustainable use of forest resources in Central Africa^44^. This makes it important to map the local understanding of forest land uses, and to reconcile these with the other land uses in the region to better inform decisions concerning land use policy and planning. The objective of this research was to understand the distribution of lands currently used for wild meat hunting by 10 Baka villages in southeastern Cameroon. In this paper, we use a combination of participatory methods and monitoring of hunters to: (1) identify the communal hunting territory of each village, (2) determine whether wild meat extraction levels per village were related to the size of hunting territories utilized; (3) assess whether certain habitats were selected for hunting in the different villages and (4) determine the overlap between hunting territories of each village and extractive industries in the region.

## Study area

**Figure 1:**
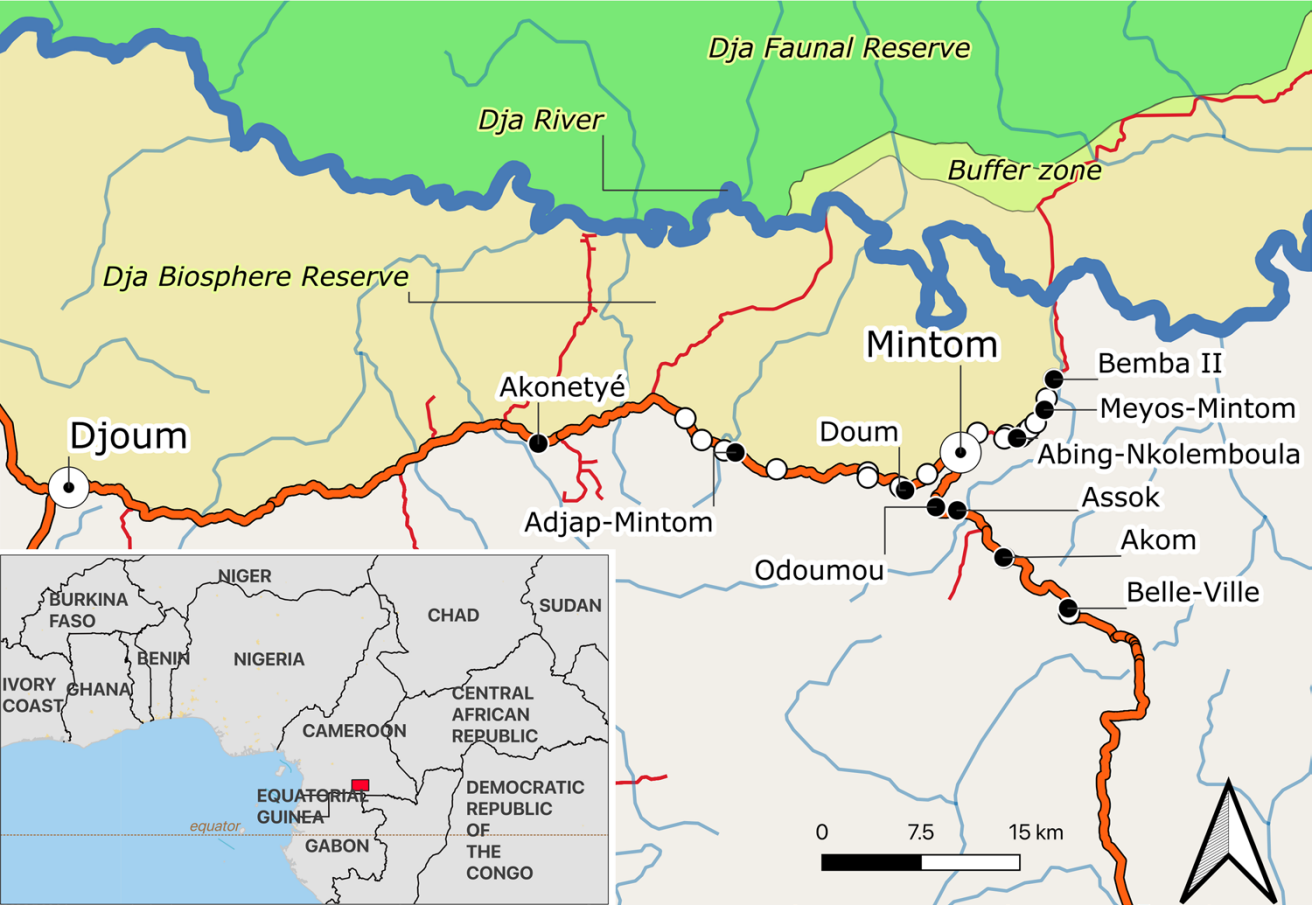
Map of the study area indicating the location of the regional cities Djoum and Mintom, the 10 Baka study villages (black circles) and neighbouring Bantu-speaking farmer villages (white circles) in southeastern Cameroon. Only villages between Akonetyé in the West, Bemba II in the Northeast and Belle-Ville in the Southeast are shown. The map was created using QGIS version 3.16.0-Hannover (https://qgis.org/en/site/) from public domain map datasets from Open Street Map (www.openstreetmap.org), diva-gis (diva-gis.org) and Natural Earth (http://www.naturalearthdata.com) and the published map of the Dja Biosphere Reserve^89^.

This study was conducted in 10 Baka villages, located along the Djoum-Mintom road south of the Dja Faunal Reserve (DFR) and bordering the Dja Biosphere Reserve in southeast Cameroon (Fig. 1). From population censuses conducted by us in all 10 villages (Table S1), we counted a total of 237 dwellings (largely mud-lattice “poto-poto” houses but also traditional “mungulu” leaf huts) of which 172 (72.57%) were lived in during the study. There was a mean (± SD) of 4.33 ± 2.77 (range 1–17) persons (men, women and children of differing ages) living in the occupied dwellings. Vacant dwellings belonged to families that were in forest at the time of the study. Total village population sizes ranged from 25 persons in Meyos-Mintom to 111 in Akom. Villages exclusively inhabited by Bantu-speaking farmers were also found on the Djoum-Mintom road (Fig. 1).

Agriculture alongside the harvest and trade of non-timber forest products are practiced by the Baka and also by the Bantu-speaking farmers but to different degrees. These groups primarily grow crops such as plantain, banana, and cassava. Such subsistence farming has increased in recent years in some of our study villages as a result of agricultural training schemes initiated by our partner Zerca y Lejos (ZyL)^45,46^, a Spanish NGO working on development and health support to Baka communities in the region. ZyL’s agricultural programme, implemented since 2018 in five villages (Assok, Akom, Bemba II, Doum, Abing-Nkolemboula) have resulted in training families in the planting and growing their own subsistence crops. Although amounts produced are still limited, an immediate effect has been a fall in Baka men having to work directly for the Bantu-speaking farmers to acquire agricultural foods. Plantain, banana, and cassava are the main food crops produced by the two ethnic groups with cocoa comprising the principal cash crop for the Bantu-speaking farmers. In all our study villages, animal protein was always obtained from hunting wild animals, practiced primarily by men but women also hunt around their farms^47^. Some hunted animals are sold mostly to neighbours^47^.

Our discussions with hunters in all of our study villages suggest that the forest spaces exploited by the village are open to all hunters in the village. The boundaries of these village hunting territories are used by every hunter but exclusive hunter territories are not the norm, since individual hunting territories used by the Baka are essentially determined by a hunter’s experience of the landscape and knowledge of its resident fauna, and by unwritten rules of respect of other hunters in the area e.g. a hunter will not set snares where snares are already set. Bantu-speaking farmers also hunt in the same areas as our Baka hunters though we did not monitor their movements of activities. Overall, Bantu and Baka hunters from adjacent villages, if they occur in forest, are non-confrontational. However, the main problem is when outsiders, especially large well organised hunting parties, enter Baka village hunting areas without permission.

No traditional hunting areas are officially recognised for the exclusive use of Baka in Cameroon. The use of forest resources in Cameroon is regulated by the 1994 Forest law and the 1995 application decree in (1) land allocated to forest or wildlife habitat and, (2) non-permanent forest, which includes “land available for a variety of uses”^48^. The practice of traditional hunting is allowed as long as the hunted or captured animal is strictly intended just for household consumption. Those who wish to hunt for commercial purposes must pay taxes, must have also sought and obtained permission or a hunting license from the administration (Law of 1994, art. 88–89). This means that in no case can game meant for subsistence be sold. Similarly, the law prohibits the traditional hunter from transporting game, even with the intention of consuming it himself or for the consumption of members of his family living in a neighbouring village or town. Thus, all products derived from the forest under the right of use in general and traditional hunting, in particular, must be consumed within the territorial limits of the vicinity of the forest^49^. Traditional hunting is strictly prohibited in protected areas (1994 Law, Articles 8 and 81), takes place throughout the year by both Indigenous groups and Bantu-speaking people, and is allowed for Class C species only listed in the Cameroon’s 1994 Forestry and Wildlife law. Class C species are partially protected; their capture and killing being regulated by the Minister of Environment and Forests to maintain the dynamics of their populations.

The terrain of the region is low-relief with gently rolling hills of a maximum elevation of 800 m, averages of 600–650 m. The climate of the region is characterized by a four-season equatorial climate. A major dry season is from December to March, a minor rainy season from March to June, a minor dry season in August, and the major rainy season from September to December. Rainfall recorded for Djoum averages 1500–2000 mm per year, and some precipitation is common even during the dry seasons^50^. Average temperature remains fairly steady year-round, averaging 25 °C, fluctuating slightly with the seasons. The major rainy season is the coldest time of year, and the major dry season the warmest. Humidity is high and does not vary throughout the year.

The major vegetation type is a mixture of evergreen and semi-deciduous forests^51^. The forest is degraded alongside the dirt roads and main road, due to pressure from housing and agriculture. Commercial timber extraction operations in the region, which started in the 1970s, have enabled the penetration of intact forest and have increased agricultural and hunting pressures, facilitated by newly created logging roads^52,53^. These roads now connect our study villages to market areas and to the main towns of Mintom and Djoum (Fig. 1). There is evidence that logging operations have negatively impacted both the natural environment as well as Baka livelihoods through deforestation, along with the construction of roads and logging activities^10,13,54^. Wildlife habitats have also been affected. At the same time, a huge influx of logging labourers and an expansion of the wild meat trade has endangered some wildlife species threatening not only Pygmies’ daily lives (which highly depend on natural forest resources in terms of diet and medicine), but also their cultural identity, which is closely tied to the forest^55^. In addition, logging operations tend to harm relationships between Pygmies and neighbouring farmers^56^.

## Permits and ethics

Permission to undertake field work was granted by the Cameroonian Ministry of Scientific Research and Innovation (MINRESI), via the nationally represented Center for International Forestry Research (CIFOR). Authorization to work with human subjects was covered by Arrete No. 00034/A/MINATD/DAP/SDLP granted by the Ministère de L’Administration Territoriale et de La Decentralisation of the Government of Cameroon to ZyL. We followed the principle of free, prior and informed consent (FPIC) in which all hunters in our study freely participated in our project. We obtained informed consent from all participants who could stop contributing to the project if they so wished^57^. Participants were made aware that their decision to participate or not would have no negative consequences. They were also informed that their identity would be kept anonymous and all personal information provided would be treated confidentially. Our study also followed the ethical research guidelines set out by the Social Research Association. Although children were present in the participatory workshops because these meetings were open to everyone in the villages (see below), no one under the age of 18 (minors according to Cameroon law) was directly involved in our study.

## Data collection

### Hunter offtake

Information on animals hunted was available from a total of 1946 hunting trips undertaken by 122 hunters from the 10 study villages. This information was recorded during our Darwin project^58^, between March and July 2018 in Abing-Nkolemboula, Assok, Belle-Ville, Bemba II and Doum, and from Oct. 2018 to Feb. 2019 in Adjab-Mintom, Akom, Akonetyé, Meyos-Mintom and Odoumou. In each village, we recruited a village reporter (VR) to oversee data collection from participating hunters in the village. Hunters provided daily information to a village reporter on the type of animals hunted, hunting method used, start time and end time of each hunting day and whether the quarry was to be consumed in their home, the complete carcass sold, or parts of the animal consumed, and other parts sold. Complete results from this study have been published elsewhere^47^; here we used the mean number of carcasses successfully extracted per hunter (Table 1). All hunting tracks were gathered for a subset of hunters for which offtake information was collected in all the 10 villages. Because offtake data were collected on a village basis, it was not possible to match hunting trips for offtake information and track characteristics.

**Table 1:** Distances and velocities covered by hunters during hunting sessions in 10 Baka villages.

Village	Hunter movements				Offtake data		
	# Hunters	# Tracks	Mean track distance [km]	Mean velocity [km h^−1^]	# Hunters	# Carcasses	Offtake [carcasses hunter^−1^]
Abing-Nkolemboula	8	46	20.7 ± 12.4	1.8 ± 1.7	12	271	22.58
Adjap-Mintom	5	21	12 ± 8.2	1.4 ± 1.2	8	101	12.63
Akom	5	23	10.4 ± 5.2	1.2 ± 0.6	9	399	44.33
Akonetyé	7	30	8.3 ± 2.6	0.8 ± 0.3	13	162	12.46
Assok	4	17	22.7 ± 6.3	1.4 ± 0.6	12	94	7.83
Belle-Ville	4	35	8.2 ± 2.5	1.4 ± 0.7	13	135	10.38
Bemba	5	20	18.3 ± 6.9	1.3 ± 0.5	22	307	13.95
Doum	4	47	24.7 ± 18.4	2 ± 1.3	11	382	34.73
Meyos-Mintom	5	35	19.6 ± 19.9	1.3 ± 0.8	9	231	25.67
Odoumou	4	21	14.5 ± 11.3	1 ± 0.3	13	161	12.38
∑	51	295			122	2243	
Minimum			0.9	0.02			7.83
Maximum			89.8	7.5			44.33

Offtake data of 121 hunters have been published by Ávila Martin et al. (2020) and one additional hunter was added here.

### Participatory mapping of hunting territories

To understand the issues affecting hunting and to map each Baka village’s hunting territories we organized participatory mapping workshops in every village. Before we started these workshops, we (E.A.M., G.R.B., R.O.) organized several introductory meetings in each village where we formally communicated the overall aims of our project to the village chief and the community. Following the principles of FPIC (see above) we explained the role each contributor would have in the project. During these meetings, we first asked the participants to highlight the main challenges faced in the daily lives; most identified key problems around agriculture and hunting issues.

Workshops were managed in two separate phases (05–22 Dec. 2017: Assok, Belle-Ville, Bemba II, Doum, Abing-Nkolemboula; 19 Sept. 2018–19 Oct. 2018: Adjap-Mintom, Akonetyé, Akom, Meyos-Mintom, Odoumou). Workshops were open to all villagers (men, women and children) and lasted around 5 h; a total of 53.08 h for the 10 villages (Table S2). On the eve of each workshop, we held an initial contact meeting during which we consensually arranged a time for the planned exchanges; we chose the time that best suited the community, early in the morning. All workshops, undertaken in Fang, the *lingua franca* between the Baka and the local Bantu-speaking farmers, were led by two members of our team (R.O., F.F.). Our team were assisted by three local facilitators (Jean Ndoutoumou, Luc Ndeloua and Blaise Ango Ze), respected Baka elders who had been hunters themselves and who worked as schoolteachers with ZyL, hence literate as well as proficient in French, Fang and Baka. Their role was to help participants understand the exercise at hand, and interpret from Baka to Fang, when needed.

We introduced the aims of the exercise at the start of each workshop. Hunters who participated in the workshops were then asked to denote the areas where they undertook activities important in their lives, or of significance for their wellbeing. Participants were asked to mark on a 1:50,000 A-0 size base paper map containing the main cartographic features of the region which areas were crucial for their livelihoods e.g., “terroirs” and activity zones (*Mokongo*), hunting trails (*Pkwa djé*), points of cultural value, rivers used for fishing, old villages (*Di bala*), hills (*Kenga*) as well as areas of primary forest (*Woulou*) and campsites. During all workshops, participants were encouraged to examine and validate the resulting participatory maps, as well as to discuss issues such as the status of game populations in their hunting territories. However, for the present paper, we only used the areas delimited by the hunters as the hunting territory for their village, and no other. We also included camps located by the participating hunters on our maps, see^11^. These camps, which are used as a base for hunters when staying in the forest for more than a day, are often located in favoured spots in the forest, typically on a rise beside a stream. All maps were first digitalized and then included in an ArcMap 9 GIS as separate shapefiles for further spatial analyses. Photographs of three of the original maps as examples are shown in Fig. S1.

### Monitoring of hunter movements

We invited hunters who attended the participatory mapping workshops to join in mapping their hunting trips. Two GPS Garmin Foretrex 401 were allocated to each village. The Garmin Foretrex 401 features a high-sensitivity GPS receiver with hotfix for improved performance and reception in heavy tree cover or deep canyons. Prior to allocating to the villages, we tested the Foretrex GPS units in the hunting areas where they would be recording. The Foretrex does not record dilution of precision or some similar metric by which to assess accuracy throughout track recording. However, in direct comparisons of location accuracy (i.e., accuracy given on the unit screen and location coordinates) they performed comparably to a hand-held Garmin 62 which is equipped with a large antenna, and accuracy was consistently < 10 m, apart from a few instances under very dense canopy where signal was lost completely but this only occurred very sporadically and was not long in duration. Hunting tracks were gathered during the same period as the hunter offtake data.

Hunters who agreed to join our study were issued with the village GPS by the VR (see above) in a previously agreed month for an entire month. Hunters were free to record whichever trip they wanted to, during their allocated month. For each recorded trip hunters would collect the GPS from the VR in the morning and return this at the end of their tour. Hunters were given 500 CFA (0.83USD) as a small incentive for recording each trip.The GPSs were set to record locations every 90 s. Hunting tracks were analysed on an individual and village level. Coordinates collected as longitude–latitude in degrees by the GPS Garmin Foretrex 401 were transformed into the UTM coordinate system for further analysis and mapping. The straight distance from each of those points to the village centre in meters was calculated in ArcMap 9.

### Forest cover classification and forest use maps

A simple remote classification of forest cover type was conducted over the study area so we could attribute hunting areas to a habitat type. We classified forest cover using elevation data and satellite imagery as the base input data; elevation data was obtained from the global Shuttle Radar Topographic Mission (SRTM) available globally as spatially explicit raster data at 30 m resolution, accessed and downloaded via the Remote Pixel satellite data web portal^59^. European Space Agency Sentinel-2 satellite imagery was downloaded at a 20 m resolution from the Sentinel-hub web resource^60^. An unsupervised classification was then conducted with SAGA-GIS geoscientific analyses software^61^ using the SRTM data to account for elevation, and Sentinel-2 bands 9 and 12 to account for wetness of habitats. A total of 12 different vegetation classes of similar spectral and elevation classes was obtained. We grouped these classes into wet (swamp), lowland and upland forests based on field observations of our field team. Forest cover types were subsequently intersected with the participatory maps and hunting track kernels to determine the different forest types used for hunting by each village.

To determine the areas of overlap between extractive industries and hunting territories we used Global Forest Watch forest management unit maps^62^ and mining concessions^38^. Community forest areas were also derived from maps in Global Forest Watch^63^. All Global Forest Watch maps were updated in 2018.

## Data analyses

Hunter home ranges were estimated using the minimum convex polygon, MCP, and the kernel space use estimation methods^64^. An MCP encloses all locations with the smallest possible polygon whereby all angles of polygon segments are equal or less than 180° and no line between any two points falls outside the polygon boundaries. By stepwise including greater percentages of tracking points, i.e., GPS bearings at 90 s intervals along the hunting tracks, it is possible to ascertain whether the collected tracks are sufficient to estimate the home range size such that an addition of new tracks has little or no impact on the size estimation. The kernel method estimates not just boundaries of a home range but computes utilization distributions which provide a more informative picture of land use than other estimation tools^65^. Core areas of home range use can be determined, typically defined as a 50% kernel, which are repeatedly visited and more intensively used^66^. We calculated 99%, 95% and 50% kernels. Preference or avoidance of wet, midland and upland forest habitats for in the 50% kernel areas compared to the available habitats in the total participatory hunting areas were estimated using Ivlev’s electivity index *E*^67^. It measures the utilization of a resource, in this case habitats, relative to their availability in the environment whereby *E* ranges from − 1 for total avoidance to 1 for total preference:

$$ E = \, \left( {r_{{\text{i}}} - p_{{\text{i}}} } \right)/\left( {r_{{\text{i}}} + p_{{\text{i}}} } \right) $$

where *E* is the measure of electivity of a habitat type, *r*_i_ is the sum of all habitat types and *p*_i_ the relative abundance of a habitat.

To describe the pace at which hunting trips were conducted a mean velocity was calculated at distance travelled h^−1^ for each trip. Total hunting track lengths and home range sizes were compared with hunter’s and village’s hunting offtake data. We investigated the relationship between hunter offtake and track lengths for those hunters where both parameters were known using generalized linear mixed modelling, GLMM. GLMM was used instead of generalized linear models, GLM, in order to account for the data grouping of multiple trips per hunter and multiple hunters per village.

All statistical analyses were performed in the R statistical environment^68^. The R package ‘adehabitatHR’^64^ was utilized for home range analyses. Jarque–Bera normtest for testing normality of the data^69,70^ were performed. The non-parametric Kruskal–Wallis test was used to examine whether distances travelled come from village groups with equal medians. GLMM was conducted with the R package ‘lmerTest’^71^ for normally distributed data (i.e. linear mixed model) by restricted maximum likelihood, REML.

## Results

### Village hunting areas

A total of 424 community members (150 men, 135 women and 139 children) from the 10 villages attended the 10 workshops (Table S2). Most participants were Baka, but 46 (10%) were Bantu that lived in the villages or were visiting at the time of the mapping exercise.

**Figure 2:**
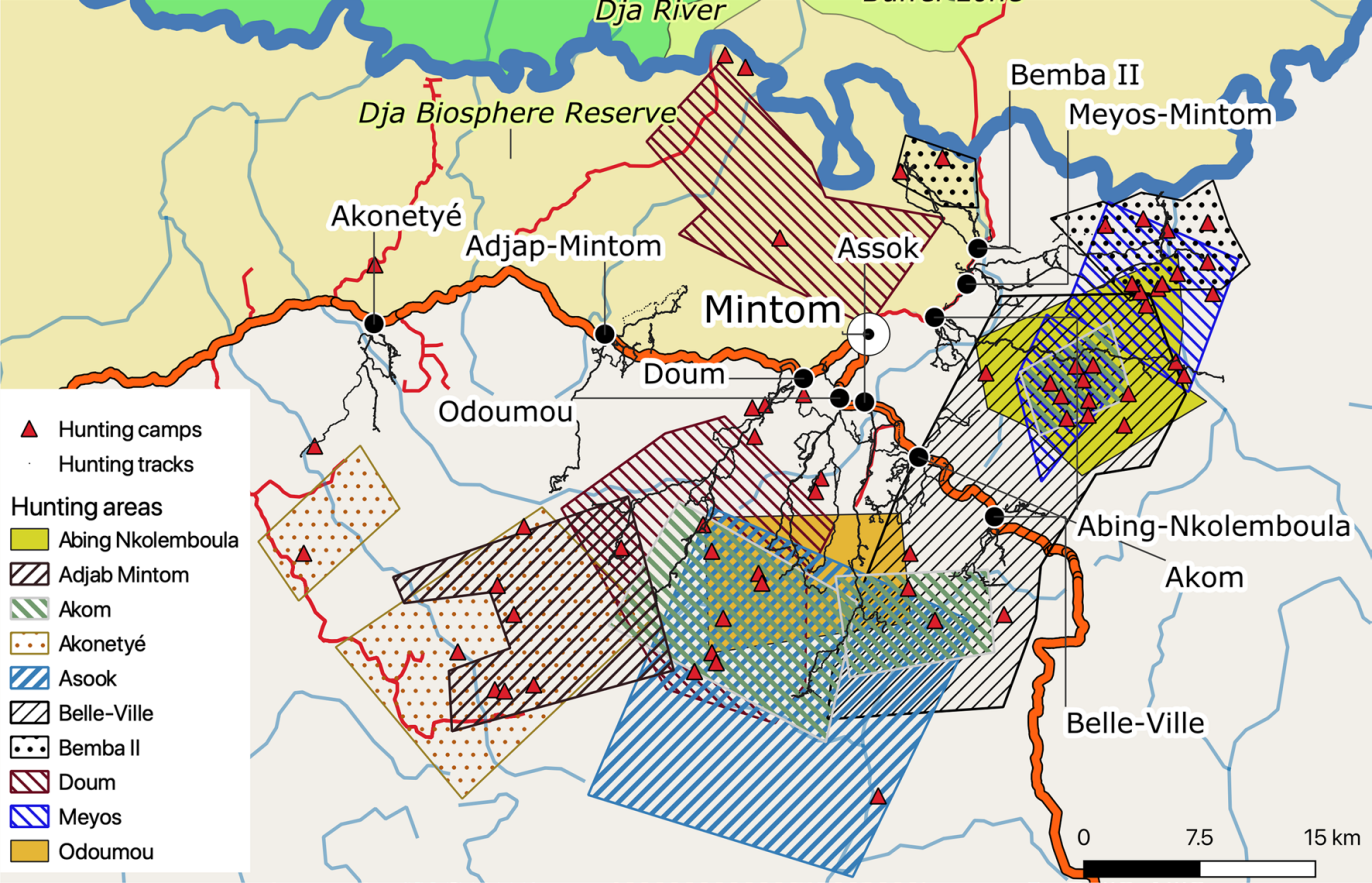
Participatory mapping territories and hunting tracks for the 10 Baka study villages in southeastern Cameroon. The background map was created using QGIS version 3.16.0-Hannover (https://qgis.org/en/site/) from public domain map datasets from Open Street Map (www.openstreetmap.org), diva-gis (diva-gis.org) and Natural Earth (http://www.naturalearthdata.com) and the published map of the Dja Biosphere Reserve^89^.

Hunting areas were delimited by the participating hunters on the basis of their knowledge of landscape features. Mapped village hunting areas averaged 205.2 ± 108.7 km^2^ (ranging from 76.8 km^2^ in Bemba II to 352.0 km^2^ in Doum), a total of 2052.4 km^2^ across the 10 villages (Fig. 2, Table S3). The average overlap between participatory hunting areas of all villages was 15 ± 25%, ranging from no overlap between villages in 51 out of the 90 pairs to 100% in only one pair (Table S3). We recorded a total of 59 camps, most (n = 50) fell within the village hunting areas; but hunting areas were unrelated to the location of the camps.

### Hunting trips

**Figure 3:**
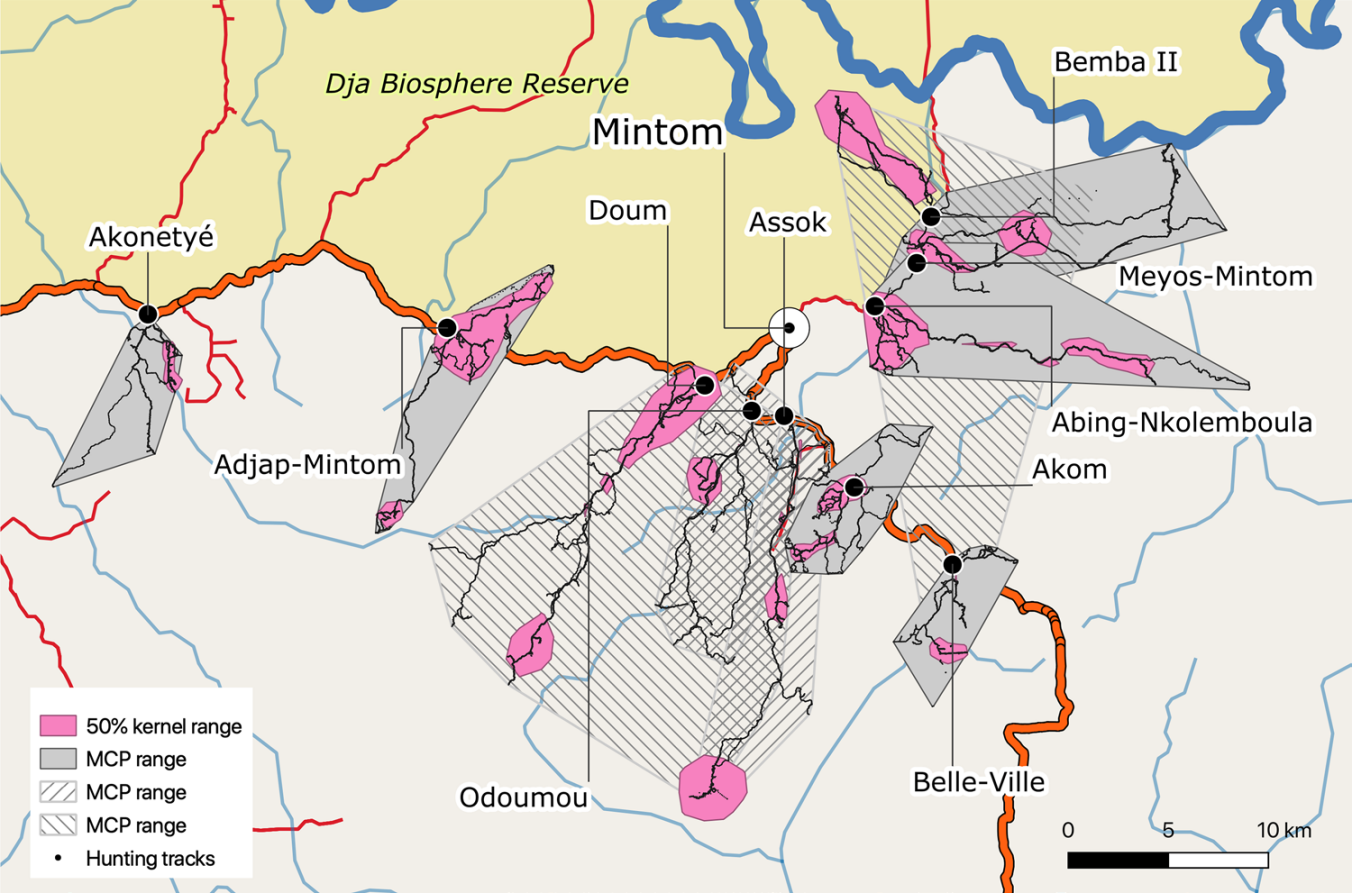
Hunting tracks, MCP home ranges and 50% kernel utilization distributions for hunters in the 10 Baka study villages in southeastern Cameroon (295 tracks recorded for 51 hunters). The background map was created using QGIS version 3.16.0-Hannover (https://qgis.org/en/site/) from public domain map datasets from Open Street Map (www.openstreetmap.org), diva-gis (diva-gis.org) and Natural Earth (http://www.naturalearthdata.com) and the published map of the Dja Biosphere Reserve^89^.

Between four and eight hunters per village participated in tracking hunter trails in the 10 villages, resulting in 295 tracks recorded for 51 hunters (Table 1). Each hunter logged on average 5.8 hunting tracks (range 1–22, median 5). All tracks are shown in Figs. 2, 3 and 6. Assuming one hunter every occupied household (from data in Table S1) we estimate that an average of 38 ± 27% (17–100%) of all hunters in each village participated in monitoring hunting trips.

**Figure 4:**
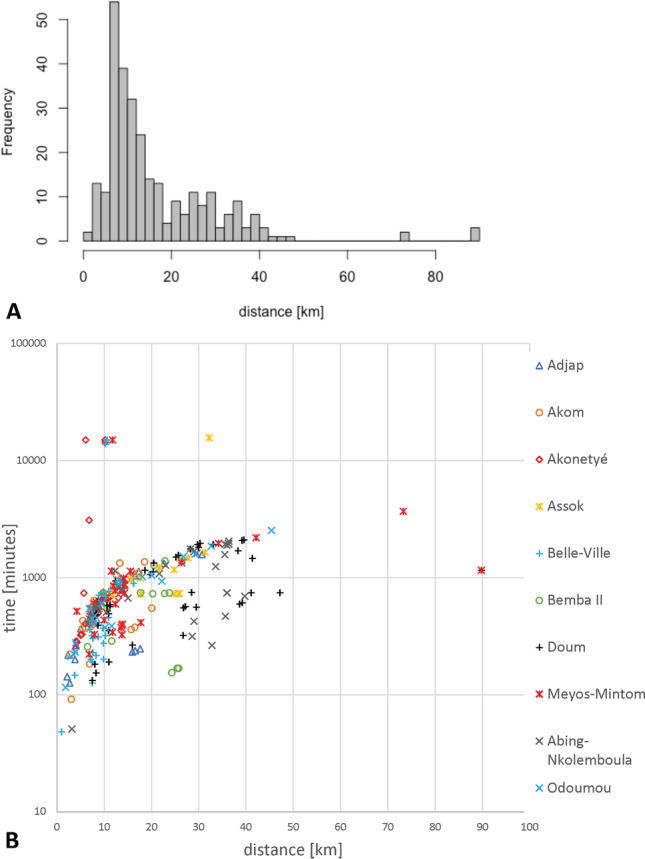
Track distances for 51 hunters in the 10 study villages in southeastern Cameroon (**A**) and the relationship between distance and time for each recorded hunter track by village (**B**).

Average distance travelled by a hunter in a hunting trip (Table 1, Fig. 4) was 16.5 ± 13.5 km (range 0.95–89.8 km), with furthest average distances recorded in Assok (mean 22.7 km) and shortest in Belle-Ville (mean 8.2 km). Hunting trips were conducted at a relatively slow pace, a mean velocity of 1.4 km h^−1^ (range 0.02–7.5 km h^−1^). Mean distances were left-skewed across all hunters and trips (Jarque–Bera test for normality, T = 2.47, *p* < 0.001). However, distances were not equally distributed across villages (Kruskal–Wallis χ^2^ = 82.9, df = 9, *p* < 0.001). In Akonetyé, movement patterns were markedly different from the more straight, unidirectional patterns of the other villages (Fig. 3). Distances were particularly large for the villages of Adjap and Assok, which were characterized by the most unidirectional trails.

### Village hunting ranges determined from hunter trips

Figure 3 shows the hunting tracks, minimum convex polygon (MCP) home ranges and the 50% kernel utilization distributions. Home range size in relation to the percentage of recorded locations are shown in Fig. S2. Hunters in some villages used different paths whereas in others hunter movements were straighter and unidirectional. Graphs of MCP home range size relative to the percentage of included tracking points showed that home range sizes did not reach a plateau (Fig. S2), indicating that the number of locations and tracks are not sufficient to estimate total home range sizes. Pearson’s correlation coefficients r between total hunter offtake in each village (n = 10) versus MCP size and 99%, 95% and 50% kernel utilization distribution size were 0.24, 0.26, 0.22 and 0.43, respectively. Pearson’s r between village total hunter offtakes versus village hunting ranges was 0.05.

### Relationship between hunter offtakes and hunting trips

**Figure 5:**
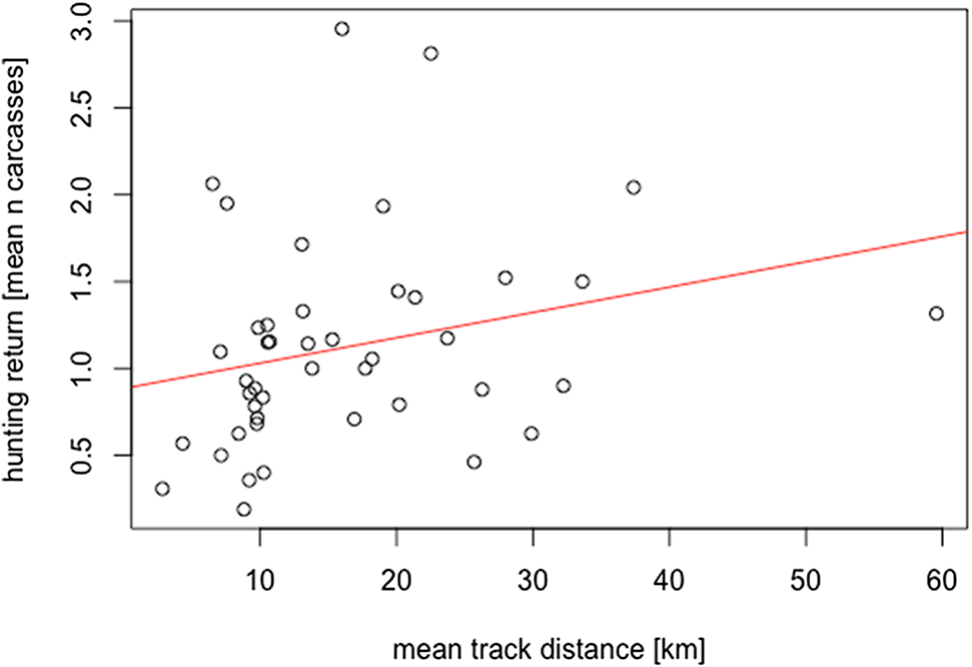
Relationship between mean track distances and hunting returns.

Offtake information was available for a total of 44 trips. For these hunters, mean travelled distance per hunting trip and mean returns were left-skewed (Jarque–Bera test for normality, T = 1.82, *p* < 0.001, and T = 1.13, *p* = 0.003, respectively), but the log-transformed data were normally distributed (T = 0.026, *p* = 0.94, and T = − 0.52, *p* = 0.012, respectively). There was a trend towards a significant higher offtake at greater distances covered (Fig. 5, linear mixed model fit by REML of log-transformed data, n = 44, village groups = 10, t = 2.7, *p* = 0.009).

### Habitat correlates of hunter territory use

For all villages pooled, the average hunting home range core areas, as estimated by the 50% kernel method, was 73.4 ± 5.9 km^2^ (Table 2). Forest types were very unequally distributed between villages home range core areas ranging between 1 and 96% for wet forests, 3 to 54% of midland forests and 1 to 64% of upland forests. Habitat availability in the total participatory hunting area (Fig. S3, Table 2) was at a similar range for wet forests (1–97%) and midland forests (1–51%) but varied even more widely in upland forest (2–95%). Ivlev's electivity index *E* indicates that midland forests were selected for core home range areas more than their overall availability (mean *E* = 0.36 ± 0.43 across all villages). Wet and upland forests were on average selected less or avoided as their values were closer to zero (mean *E* = 0.06 ± 0.38 and − 0.13 ± 0.41, respectively). On a village level, habitat selection appears more pronounced. In Bemba II, for example, rare upland forests were avoided (*E* = − 0.6), rare midland forests preferred (*E* = 0.71) and the very abundant wet forests neutral (*E* = − 0.01).

**Table 2:** Use of different habitat types during hunting sessions in 10 Baka villages.

	Hunters	Tracks	50% Kernel utilization distributions				Participatory hunting area				Ivlev's electivity index E		
			∑ [km^2^]	Wet forest [%]	Mid forest [%]	Upland forest [%]	∑ [km^2^]	Wet forest [%]	Mid forest [%]	Upland forest [%]	Wet forest	Mid forest	Upland forest
Abing-Nkolemboula	8	46	16.9	85	12	3	123.4	77	5	17	0.04	0.38	− 0.68
Adjap-Mintom	5	21	14.2	13	24	64	145.1	3	4	94	0.65	0.72	− 0.19
Akom	5	23	4.3	16	54	29	219.9	41	37	22	− 0.43	0.19	0.14
Akonetyé	7	30	1.9	1	41	58	229.3	1	4	95	0.24	0.80	− 0.24
Assok	4	17	15.1	64	26	10	349.9	38	37	25	0.26	− 0.18	− 0.42
Belle-Ville	4	35	2.5	28	54	18	337.9	55	13	32	− 0.32	0.61	− 0.28
Bemba II	5	20	3.9	96	3	1	76.8	97	1	2	− 0.01	0.71	− 0.60
Doum	4	47	194.4	11	29	61	352.0	38	47	14	− 0.57	− 0.25	0.62
Meyos-Mintom	5	35	9.6	76	19	6	115.7	92	2	6	− 0.10	0.78	0.00
Odoumou	4	21	4.5	10	38	52	102.2	22	51	27	− 0.38	− 0.14	0.32
Mean ± SD			26.7 ± 59.2				205.2 ± 108.7				− 0.06 ± 0.38	0.36 ± 0.43	− 0.13 ± 0.41

### Overlap between hunting territories and extractive industries

**Figure 6:**
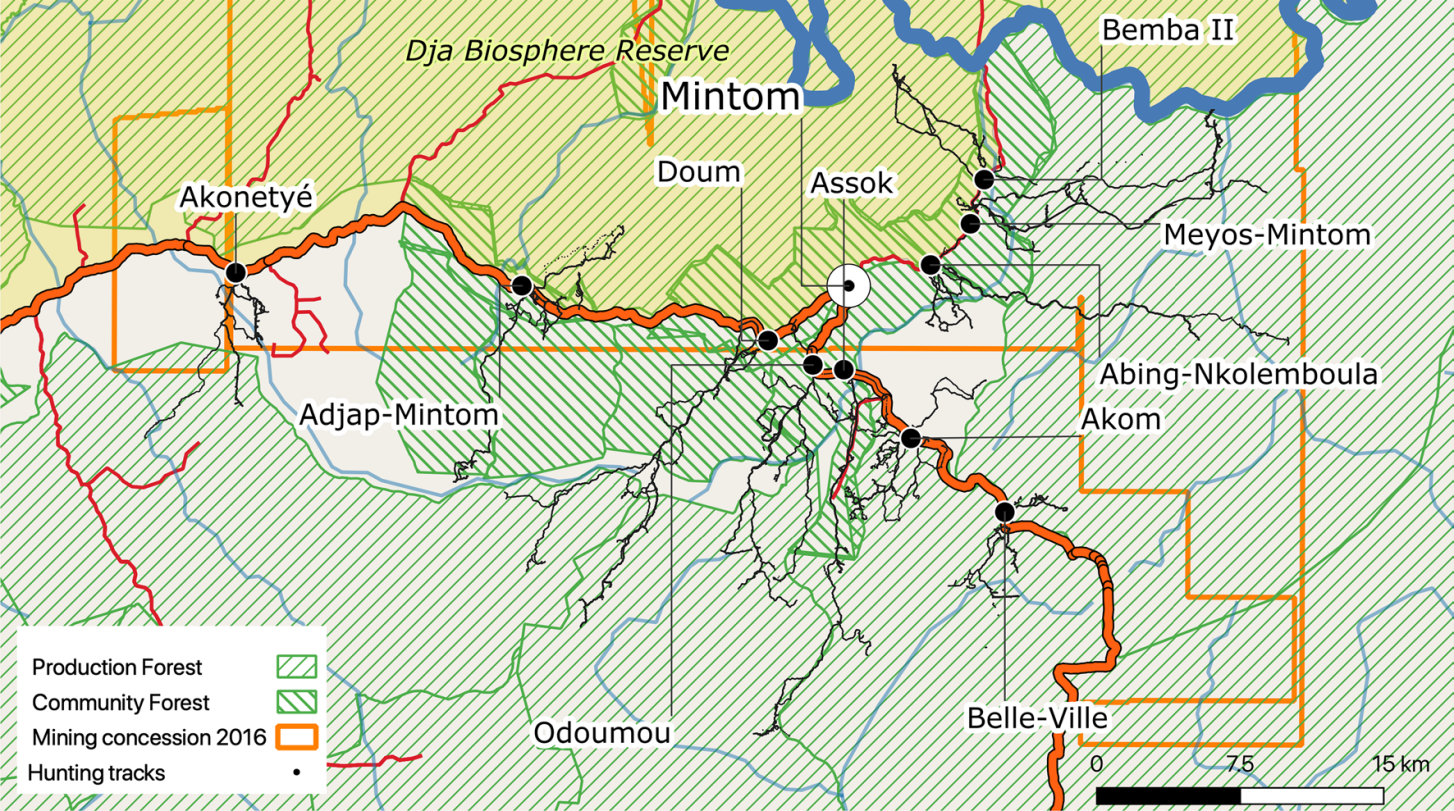
Map of overlap of hunting tracks and logging and mining concessions for the 10 Baka study villages in southeastern Cameroon. The background map was created using QGIS version 3.16.0-Hannover (https://qgis.org/en/site/) from public domain map datasets from Open Street Map (www.openstreetmap.org), diva-gis (diva-gis.org) and Natural Earth (http://www.naturalearthdata.com) and the published map of the Dja Biosphere Reserve^89^.

Figure 6 shows the overlap between logging and mining concessions and hunting tracks for all villages. All hunting areas fall within extractive industry concession zones.

## Discussion

The livelihood system in our Baka villages is based on small-scale farming centred on shifting cultivation as well as the collection of various products from the forest, including non-timber forest products, hunting, fishing, and small-scale logging for domestic use. This system is typical for other Pygmy communities in the region, especially those groups now living along roads^72,73^. The large volume of data obtained in this study was possible by engaging a large number of community members. The work reported here focused on determining spatial use though we also employ offtake data obtained in a simultaneous study to understand the relationship between hunting territory size and extraction levels.

We used widely applied techniques such as participatory mapping of indigenous lands; a technique that has been in existence since the 1960s^74^. This method can be a powerful tool for Indigenous Peoples to use to accurately delimit the areas currently used which can help these groups defend and claim their ancestral lands, see e.g.,^75^. A variety of methodologies have been used, ranging from highly participatory approaches involving village sketch maps to more technical efforts with geographic information systems (GIS) and remote sensing^76^. Alongside the communal mapping of territories undertaken in each village, we also engaged with individual hunters for them to record their movements when hunting using a wrist-held GPS. This dual approach has allowed us to demonstrate occupation and use of specific areas by the study villages as a result of the willingness of communities to engage and participate and the successful application of this approach through our team’s ability to encourage the sharing of local knowledge and experience. Because our facilitation was led by Cameroonian nationals in our team, who spoke a common language with our Baka villages, we guaranteed clarity in the delivery of the process whilst ensuring that these exercises help to better understand the potential conflict areas with other land uses in the same region. In line with the work undertaken by Njounan Tegomo et al.^77^ on Baka communities near the Boumba-Bek National Park, also in southeastern Cameroon, our study provides much needed knowledge not only for improving wildlife conservation but also for complying with indigenous rights. We are aware that participatory mapping, as we have used, may include some imprecisions when translating oral discussions to actual maps. However, although we did not test the accuracy of all locations mentioned by hunters, we were able to verify the accuracy of a number of campsites when performing other field work. Despite this, we are confident that the mapped polygons are suitable approximations where the hunters operate. The boundaries of these areas are not likely to be straight lines but they cover landscapes delimited by the hunters themselves. In most cases, hunter tracks reach the hunting areas in all villages except Akonetyé. This discrepancy may be related to the lower sample sizes for this village and not necessarily because the delimited hunting area was not used by the hunters of that village.

Our tracking data are consistent with the observation that, as subsistence hunters worldwide, see^78–80^, hunters in our study are essentially central-place foragers who cover a core hunting territory around the village of residence. A hunter normally leaves his village in the morning for a one-day walking hunt, during which he will set or collect animals caught in traps, or if possessing a gun, will pursue preferred, large-prey species. Hunters rarely undertake overnight hunting trips, but when they do they stay in already established camps; a large number of active and disused camps were mapped in our participatory mapping workshops. Depending on prey numbers killed, the hunter will then make a decision to return home. From results of a more detailed study of hunting offtake in our study villages, most hunters currently pursue game for subsistence purposes, since alternative meats are rarely available, but will sell a proportion of their quarry^47^.

Our data indicates that hunting trips were relatively short, generally less than 30 km (although trips of 90 km were recorded) and mostly lasting around one day. Hunters will typically stay close to their community or point of access to a hunting area if animals are present, as demonstrated by the fact that hunting intensity (activity) decreases with distance from the start point. In tropical environments, distances travelled by hunters vary, from less than 5 km as in the case of Panamanian indigenous peoples^81^ or hill tribes in Indonesia^82^, to up to 30 km in the case of hunters in Monte Mitra area in Equatorial Guinea (Kümpel et al., 2008). Such variation in distances reflect the state of the provisioning environment (i.e. prey abundance) and therefore is a good indicator of hunting pressure. The distance that hunters will travel is related to measures of animal encounters, animal densities, or hunting yields, providing clear evidence of local depletion of commonly hunted animals around population centres^84,85^. The relatively high Pearson’s correlation coefficient between total hunter offtake and the 50% kernel utilization distribution size likely reflects that hunters regularly revisit productive areas, where offtake is high. The 95% and 99% kernel utilization distributions contain more areas which are less often visited and this is likely because prey encounter rates are lower, thus explaining the lower *r* values of about 0.2. Not surprisingly, *r* was zero for offtake versus village hunting ranges.

Recent studies have described the spatial distribution and dynamics of hunters, hunts, prey, and hunting territories^40,41,81^, but few have analysed the causes of territory variation relating to hunters and their prey, but see^43,80^. In our study, we show that hunting territories cover a number of different habitat types in the region and that, according to our electivity index results, there seems to be an active selection of some habitat types. In our analyses of offtake^47^ we show that the proportion of reptiles hunted in Bemba II and Abing-Nkolemboula is related to the greater presence of swamp forests. This is expected since deep forest, early successional stages/fallow areas, or swidden gardens will attract different animal species. The reasons for the choice of habitats by hunters in our study villages is unknown, so further understanding of the spatial distribution of hunting pressure, faunal diversity and abundance in each habitat is necessary. Moreover, more detailed analyses of the vegetation of the study area may allow a finer understanding of habitat selection.

We delimited village hunting areas in two distinct ways, by directly tracking hunter movements using GPS recorders and from village-based participatory mapping. Village territories derived from hunter trips and participatory maps followed the same trend, though the correlation was not significant; Bemba II village had the smallest ranges and the largest were in Doum. Some participatory ranges were unexpectedly larger in Assok, and Belle-Ville relative to the hunting ranges determined using hunter GPS follows. Although ranges resulting from hunter follows were based on a significant number of trips, because we only covered a period of five months it is possible that year-round data might reveal larger ranges, perhaps closer to the sizes calculated using participatory mapping. However, participatory ranges are likely to capture larger areas than actually used for hunting, since participants were representing the total area traditionally used by the village. These areas contained an exclusive core territory to each village but also overlapped with adjacent village areas. Whether these overlapping territories are a source of conflict between villages is currently unknown, but this is unlikely given that from our individual hunter follows, no tracks actually overlap.

The areas mapped in this study are not formally plotted, i.e., they are not registered in any national cadastral system but are based on distinct social and cultural knowledge of landscapes. They include information that is often omitted from official maps such as customary land boundaries, past villages, traditional and natural resource management practices and sacred areas. Of importance, however, is the need to determine whether the pooled area used by all study villages in our study is large enough, as a comparison an area equivalent to twice the size of Hong Kong^67^, to provide most or all the natural resources (wild meat but also food and medicinal plants^87^) needed by these communities. Participatory maps should mark the beginning of a long-term process, in which our communities and supporting organizations use the data collected to advocate for the defence and promotion of their rights as well as their interests. The maps also encourage better decision-making, as well as fair and transparent forest governance.

Although the areas used by our study communities did not embrace protected areas, most hunting territories lie within logging and mining concessions, and only a small area actually is covered by community forests. The establishment of logging or mining concessions must, by law, take into account the ancestral territories of indigenous peoples in agreements between the governmental agencies and extractive companies that administer these areas. Despite this, from discussions with our study communities, there is little evidence that these agreements are clearly understood by the Baka groups. These agreements should specify the rights and obligations of the different parties, involving not only respect for the indigenous and traditional peoples’ full rights to the traditional use of their resources. The legal recognition of indigenous and traditional peoples’ collective rights to the resources they possess, even if they are in exploited lands, should be a key consideration. However, in the case of southeastern Cameroon, exclusive indigenous territories typical of other parts of the world are absent, perhaps due to government’s fear of losing control over the natural resources these areas contain. These issues are compounded by the lack of definition of property rights, and of the rights to natural resource use and administration in indigenous territories, especially for the Pygmies. In Cameroon, access to forest resources is officially governed by the Forestry Law of 1994 in which there is a sharp separation of specialized productive spaces, in contrast with the long-established customary rules. Customarily, the first clearing of forest conveys permanent use rights to a kin group and its descendants. As a consequence, almost no unclaimed land remains in the region, since all the forest land marked in the pre-colonial time, for various uses such as cropping, temporary settlement, hunting, or fishing are the objects of customary permanent claims by local people.

Indigenous peoples’ territories present both tremendous opportunities and challenges for tropical biodiversity conservation worldwide. As a most compelling example, Garnett et al.^88^ describe that Indigenous Peoples manage or have tenure rights over at least ~ 38 million km^2^ in 87 countries or politically distinct areas on all inhabited continents. This represents over a quarter of the world’s land surface and intersects about 40% of all terrestrial protected areas and ecologically intact landscapes (for example, boreal and tropical primary forests, savannas and marshes). This means that if governments (and the extractive industries they liaise with) are able to work together appropriately with indigenous peoples in the land, significant benefits for conservation, the sustainable use of natural resources, and the survival of these communities will be ensured.

## Supplementary Information

Supplementary Information.
