# Supplementary material for: Hunting territories and land use overlap in sedentarised Baka Pygmy communities in southeastern Cameroon
Source: Sci Rep. 2021 Feb 10;11:3503. doi: 10.1038/s41598-021-83223-y (PMC7876010; doi:10.1038/s41598-021-83223-y)
Supplement: Supplementary file 1 — Supplementary Information. [file 41598_2021_83223_MOESM1_ESM.docx]

**Supplementary Information**

**Hunting territories and land use overlap in sedentarised Baka Pygmy communities in southeastern Cameroon**

Julia E. Fa, Guillermo Ros Brull, Eva Ávila Martin, Robert Okale, François Fouda, Miguel Ángel Fárfan, Bradley Cain, Rohan Fisher, Lauren Coad and Stephan M. Funk

3 Tables and 3 Figures

Table S1. Population censuses carried in the ten study Baka villages, southeastern Cameroon.

|  |  |  |  |  |  |  |  |  |  |
| --- | --- | --- | --- | --- | --- | --- | --- | --- | --- |
|  |  | Houses | |  | Inhabitants per Household | | | |  |
| Village | Location | Total | Occupied |  | Mean | SD | Min | Max | Total |
| Abing-Nkolemboula | N 02°42'20''/E013°19'43'' | 14 | 12 |  | 4.92 | 3.75 | 1 | 13 | 59 |
| Adjap-Mintom | N 02°40'16"/E 013°15'13" | 20 | 15 |  | 3.6 | 2.32 | 1 | 8 | 54 |
| Akom | N 02°37'28''/E 013°19'11'' | 37 | 24 |  | 4.63 | 3.16 | 1 | 17 | 93 |
| Akonetyé | N 02°42'03''/E 013°00'08'' | 32 | 23 |  | 5.05 | 2.5 | 1 | 11 | 111 |
| Assok | N 02°39'21''/E 013°17'12'' | 36 | 23 |  | 3.3 | 2.87 | 1 | 12 | 106 |
| Belle-Ville | N 02°35'23''/E 013°21'48'' | 11 | 10 |  | 4.1 | 2.18 | 1 | 8 | 76 |
| Bemba II | N 02°44'44,5''/ E 013°21'13'' | 20 | 14 |  | 4.43 | 2.38 | 1 | 8 | 41 |
| Doum | N 02°40'16"/ E 013°15'13" | 30 | 24 |  | 4.54 | 2.41 | 1 | 13 | 62 |
| Meyos-Mintom | N 02°43'30''/E 013°20'50'' | 7 | 5 |  | 5 | 3.32 | 2 | 9 | 109 |
| Odoumou | E 02°39'32''/E 013°39'20'' | 30 | 22 |  | 4.23 | 2.93 | 1 | 11 | 25 |
| Grand Total |  | 237 | 172 |  | 4.33 | 2.77 | 1 | 17 | 736 |
|  |  |  |  |  |  |  |  |  |  |

Table S2. Details of participatory workshops undertaken in the 10 Baka villages to map hunting territories.

|  |  |  |  |  |  |  |  |  | |  | |
| --- | --- | --- | --- | --- | --- | --- | --- | --- | --- | --- | --- |
| Village | Date | Start time | End time | Duration (h) | Children | Women | Men | Non-Baka | Total participants | |  |
| Adjap-Mintom | 20/21 Sept. 2018 | 07:30 | 12:50 | 5.33 | 6 | 11 | 13 | 2 | | 32 | |
| Abing- Nkolemboula | 18/19 Dec. 2017 | 08:10 | 12:45 | 4.58 | 23 | 15 | 6 | 0 | | 44 | |
| Akom | 14/15 Dec. 2017 | 08:10 | 13:30 | 5.33 | 16 | 22 | 41 | 8 | | 87 | |
| Akonetyé | 24/25 Sept. 2018 | 08:20 | 14:15 | 5.92 | 13 | 11 | 17 | 5 | | 46 | |
| Assok | 14/15 Dec. 2017 | 08:10 | 13:30 | 5.33 | 6 | 11 | 9 | 2 | | 28 | |
| Belle Ville | 06/07 Dec. 2017 | 07:30 | 12:50 | 5.33 | 15 | 13 | 9 | 4 | | 41 | |
| Bemba II | 21/22 Dec. 2017 | 08:10 | 13:30 | 5.33 | 23 | 17 | 16 | 4 | | 60 | |
| Doum | 11/12 Dec. 2017 | 08:10 | 14:15 | 6.08 | 19 | 21 | 14 | 5 | | 59 | |
| Meyos-Mintom | 14/15 Oct. 2018 | 08:10 | 13:30 | 5.33 | 5 | 6 | 8 | 10 | | 29 | |
| Odoumou | 31 Oct./20 Dec. 2018 | 08:10 | 12:45 | 4.58 | 13 | 8 | 17 | 6 | | 44 | |
|  |  |  |  |  |  |  |  |  | |  | |

Table S3. Percentage overlap of hunting territories obtained from participatory mapping workshops in the ten study Baka villages, southeastern Cameroon. The reference villages are listed in the column; for example, 71% of Adjap-Mintom´s territory is also claimed by Akonetyé but only 55% of Akonetyé’s territory is claimed by Adjap-Mintom.

|  |  |  |  |  |  |  |  |  |  |  |
| --- | --- | --- | --- | --- | --- | --- | --- | --- | --- | --- |
|  | Adjap-Mintom | Akonetyé | Akom | Assok | Bemba II | Bell-Ville | Doum | Meyos-Mintom | Abing-Nkolemboula | Odoumou |
| Adjap-Mintom |  | 71 | 7.5 | 3 | 0 | 0 | 21 | 0 | 0 | 0 |
| Akonetyé | 55 |  | 2 | 0 | 0 | 0 | 2 | 0 | 0 | 0 |
| Akom | 8 | 3 |  | 81 | 0 | 2 | 79 | 0 | 0 | 38 |
| Assok | 0 | 0 | 41 |  | 0 | 16 | 28 | 0 | 0 | 21 |
| Bemba II | 0 | 0 | 0 | 0 |  | 0 | 0 | 46 | 10 | 0 |
| Bell-Ville | 0 | 0 | 25 | 17 | 0 |  | 0 | 17 | 31 | 5 |
| Doum | 14 | 2 | 48 | 47 | 0 | 0 |  | 0 | 0 | 27 |
| Meyos-Mintom | 0 | 0 | 63 | 0 | 0 | 100 | 0 |  | 91 | 0 |
| Abing-Nkolemboula | 0 | 0 | 24 | 0 | 6 | 0 | 0 | 56 |  | 0 |
| Odoumou | 0 | 0 | 63 | 72 | 0 | 17 | 56 | 0 | 0 |  |
|  |  |  |  |  |  |  |  |  |  |  |


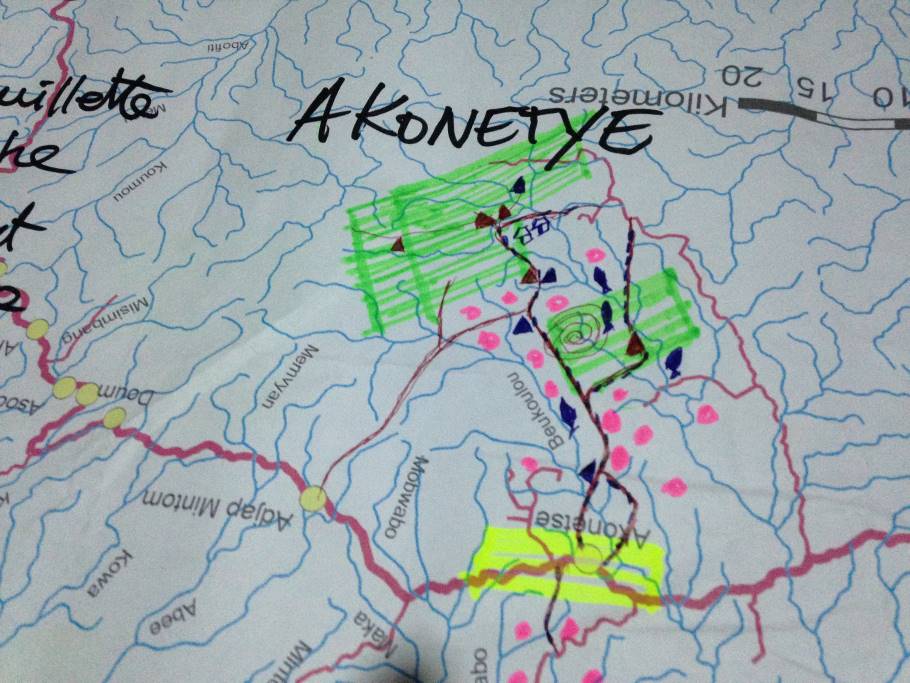


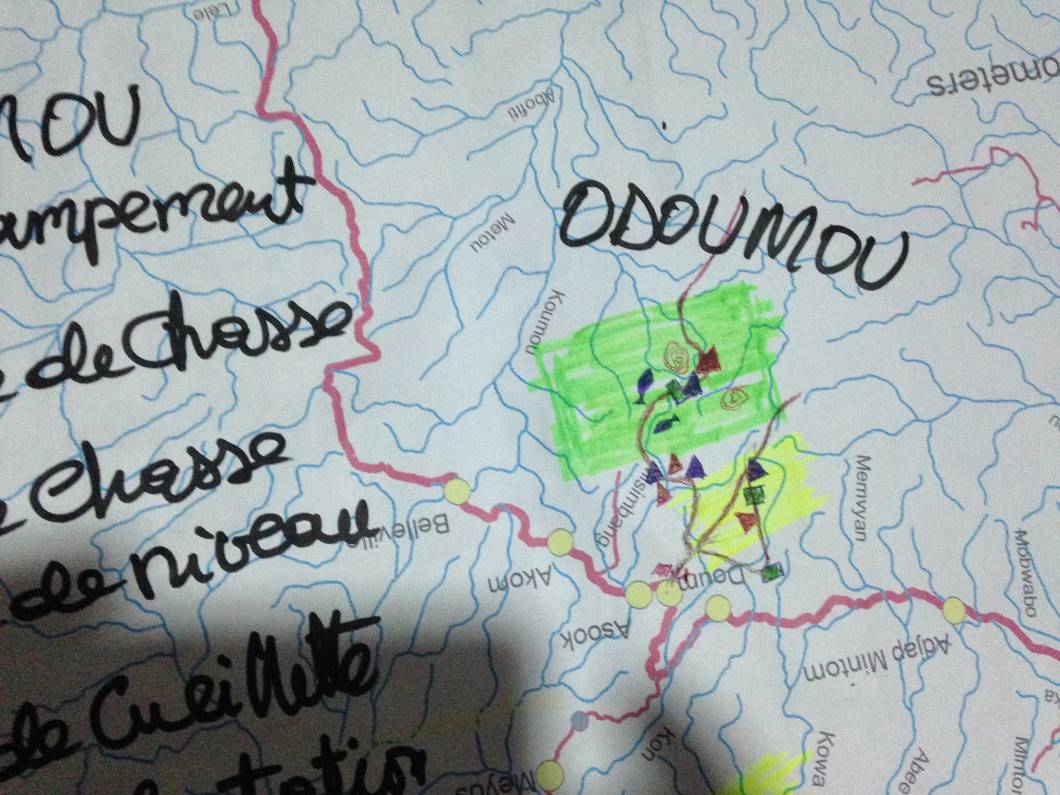


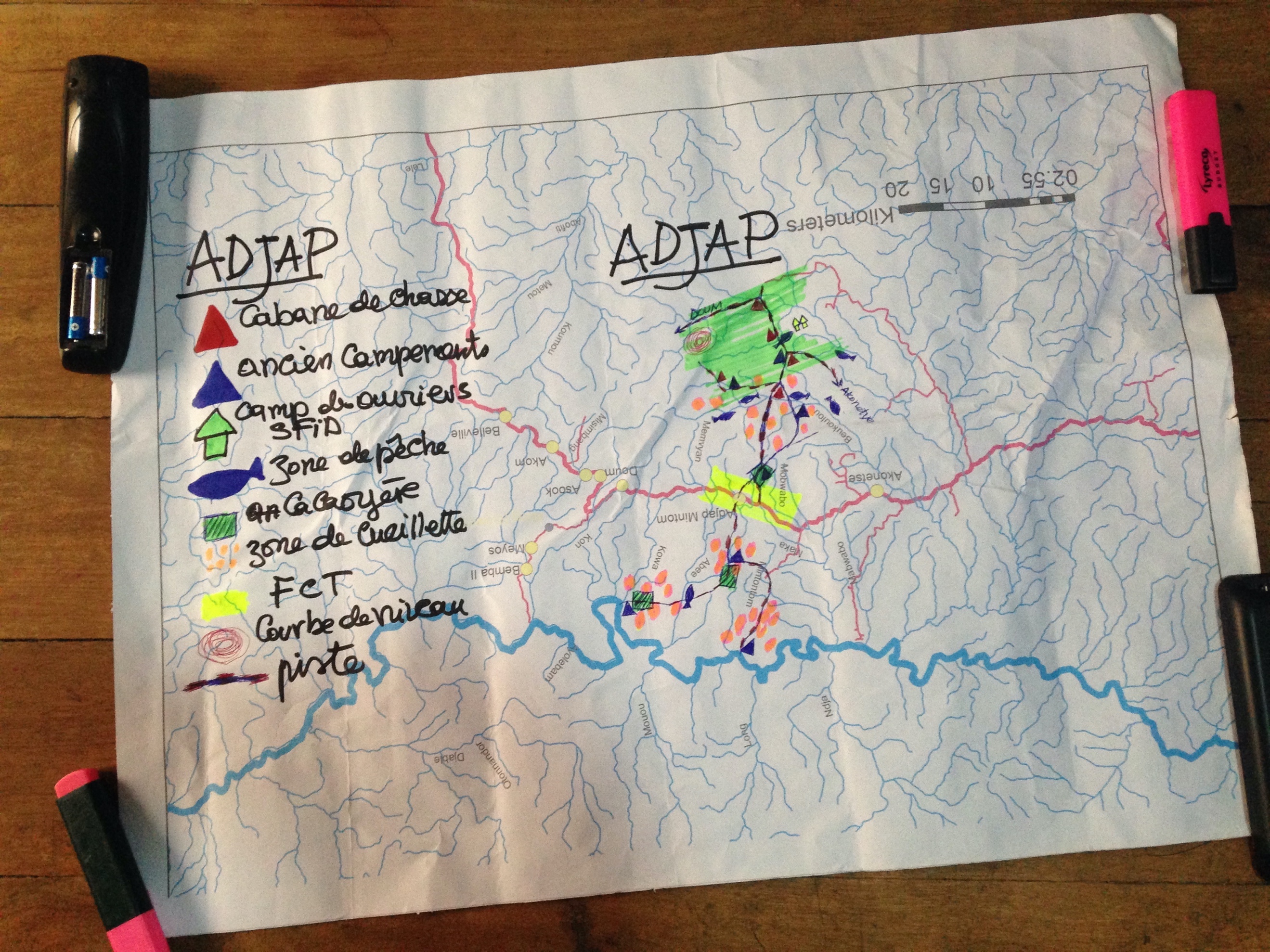


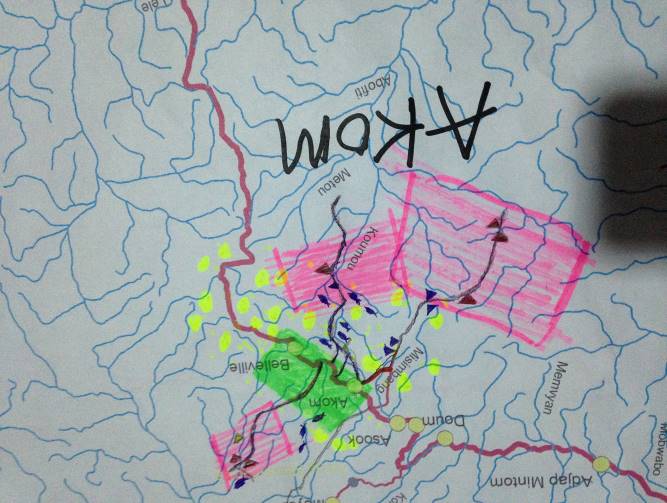


Figure S1: Examples of participatory maps resulting from workshops.


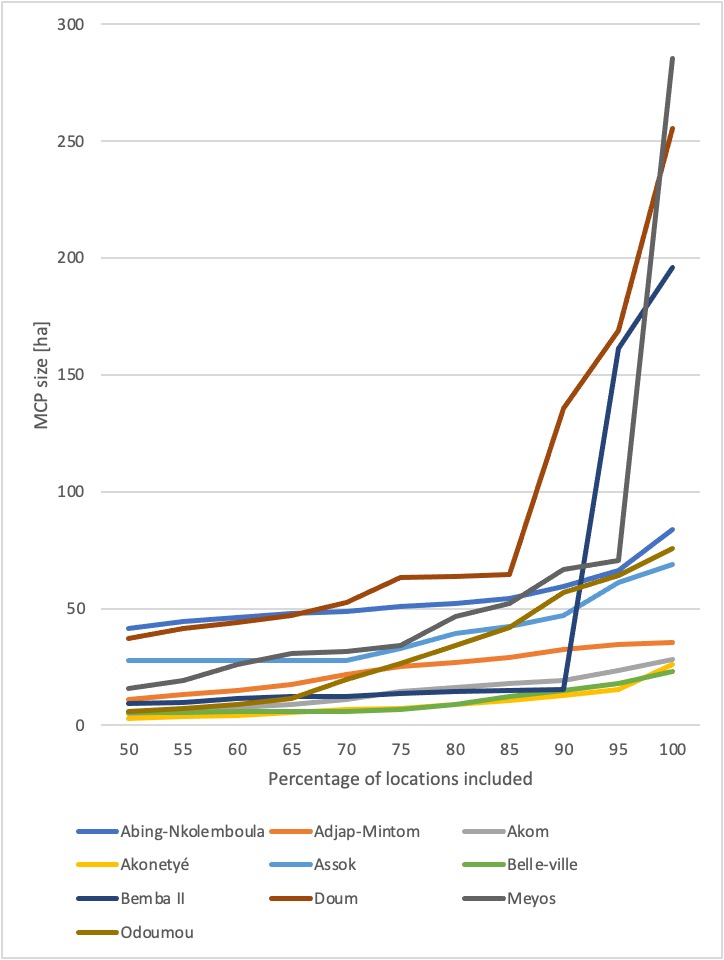


Figure S2: MCP size in relation to the proportion of localization data included in the MCP calculation.


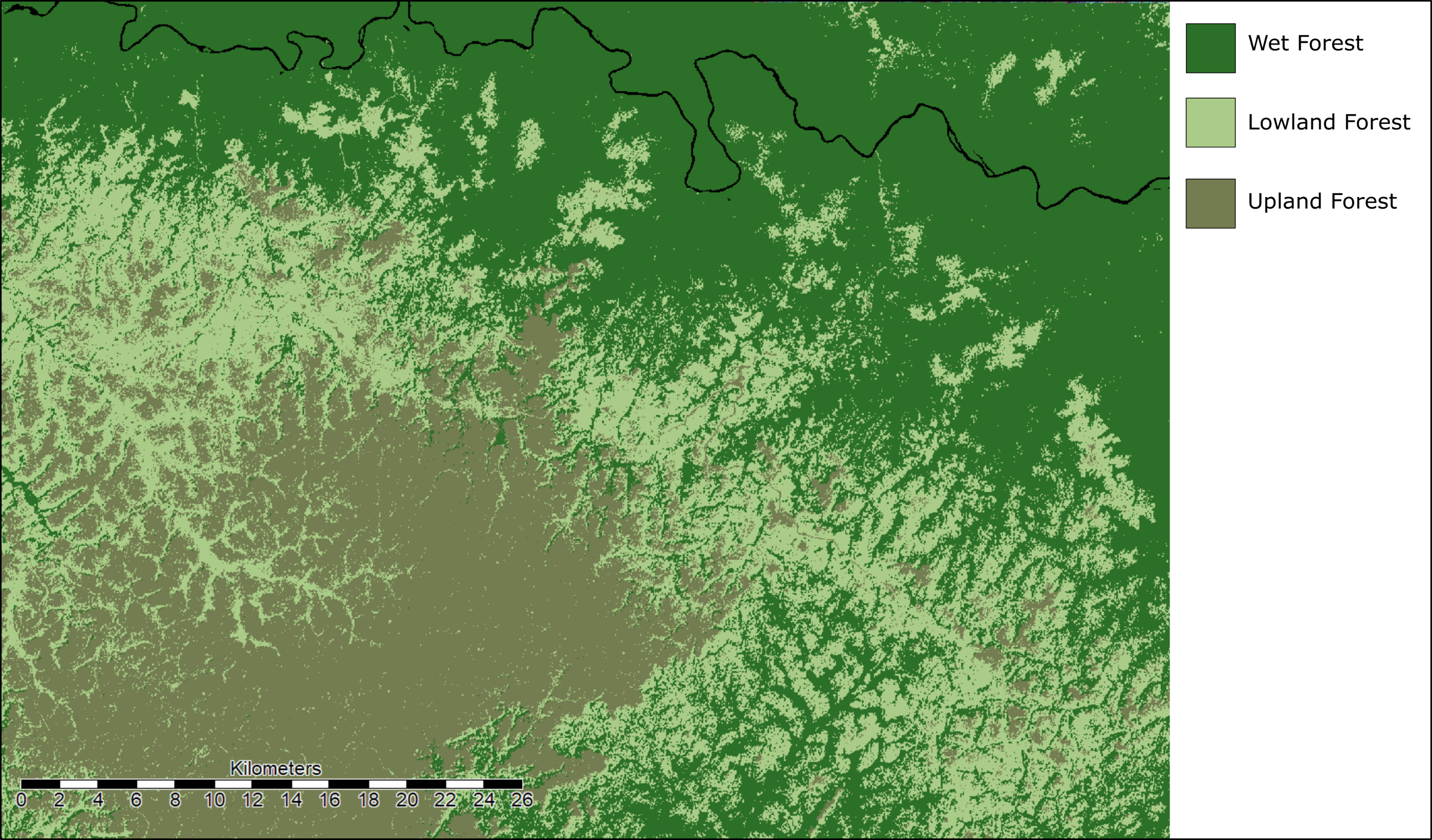


Figure S3: Forest classification map of the study area to attribute hunting areas with a habitat type. Three broad classes were used to identify primary forest cover types; wet (swamp) forest, lowland forest and upland forest. We classified forest cover using elevation data and satellite imagery as the base input data; elevation data was obtained from the global Shuttle Radar Topographic Mission (SRTM) available globally as spatially explicit raster data at 30 m resolution, accessed and downloaded via the Remote Pixel satellite data web portal^1^. European Space Agency Sentinel-2 satellite imagery was downloaded at a 20 m resolution from the Sentinel-hub web resource^2^. An unsupervised classification was then conducted with SAGA-GIS geoscientific analyses software^3^.

**References**

1. Remote Pixel. Remote Pixel - Satellite Imagery Search. <https://remotepixel.ca/> (2018).
2. European Space Agency. Sentinel online. <https://sentinel.esa.int/web/sentinel/missions/sentinel-2> (2020).
3. System for Automated Geoscientific Analyses (SAGA). SAGA - System for Automated Geoscientific Analyses. <http://www.saga-gis.org/en/index.html> (2020).
